# Manual of Medical Electricity

**Published:** 1878-12

**Authors:** 


Manual of Medical Electricity, and Illustrated Catalogue of Electrical Apparatus. Galvano-Faradic Manufacturing Co., New York.

A Summary of electrical treatment, with descriptions of the batteries, &c., used, and accounts of operations by eminent surgeons. An interesting pamphlet, and seeming new devices of merit for accomplishing the results aimed at in electrical therapy. Some of the apparatus made by this Company seems to be free from the objections commonly found. The thermo-cautery has an adjustable hand-piece, by which the current through the wire can be graduated to a nicety by a touch of the finger, instantly applied. H.

